# Live-Attenuated Oral Vaccines to Reduce *Campylobacter* Colonization in Poultry

**DOI:** 10.3390/vaccines10050685

**Published:** 2022-04-27

**Authors:** Byeonghwa Jeon, Tunchanok Saisom, Jiroj Sasipreeyajan, Taradon Luangtongkum

**Affiliations:** 1 School of Public Health, University of Alberta, Edmonton, AB T6G 2R3, Canada; 2 Department of Veterinary Public Health, Faculty of Veterinary Science, Chulalongkorn University, Bangkok 10330, Thailand; tunchanok.sai@gmail.com (T.S.); taradon.l@chula.ac.th (T.L.); 3 Department of Veterinary Medicine, Faculty of Veterinary Science, Chulalongkorn University, Bangkok 10330, Thailand; jiroj.s@chula.ac.th

**Keywords:** *Campylobacter*, live-attenuated oral vaccines, poultry, food safety

## Abstract

The control of *Campylobacter* in poultry at the pre-harvest level is critical to reducing foodborne infections with *Campylobacter* since the consumption of contaminated poultry is the most frequent cause of human campylobacteriosis. Although poultry vaccination is suggested as useful intervention measures, no *Campylobacter* vaccines are currently available. To develop live-attenuated oral *Campylobacter* vaccines, in this study, we evaluated the efficacy of pre-colonization by oxidative stress defense mutants, including knockout mutants of *ahpC*, *katA*, and *sodB*, in preventing *Campylobacter jejuni* from colonizing poultry. Interestingly, when chickens were pre-colonized with *ΔahpC* and *ΔkatA* mutants, rather than the *ΔsodB* mutant, the level of *C. jejuni* colonization was significantly reduced within 35 days. Further studies demonstrated when chickens were pre-colonized with the *ΔahpC* mutant by oral challenge with a high dose (ca., 5 × 10^8^ CFU/bird) and a low dose (ca., 5 × 10^6^ CFU/bird), it twice reduced the level of *C. jejuni* by 3.9 log_10_CFU/g feces and 3 log_10_CFU/g feces after 42 days, respectively, compared to the untreated control. Due to a colonization defect, the *ΔahpC* mutant was removed from chickens within 42 days. After excretion from the host, moreover, the *ΔahpC* mutant cannot survive in aerobic environments because of compromised aerotolerance. Our findings suggest that the *ahpC* mutant has a great potential for on-farm application to control *C. jejuni* at the pre-harvest level.

## 1. Introduction

*Campylobacter* is a leading bacterial cause of foodborne illnesses worldwide [1]. *Campylobacter* infection may develop severe abdominal cramps and watery or bloody diarrhea and is considered as the primary cause of Guillain–Barré syndrome, an acute and progressive neuromuscular paralysis [2,3]. Among pathogenic *Campylobacter* species, *Campylobacter jejuni* is most frequently implicated in human infections [4]. *C. jejuni* is a microaerophilic enteric bacterium and grows optimally at 42 °C [5]. Since the gastrointestinal tract of avian species, whose body temperatures are around 41–42 °C, provides the optimal growth conditions for *C. jejuni*–such as abundant nutrients, low oxygen concentrations, and elevated temperatures–poultry is the major reservoir for *C. jejuni*. Consequently, human exposure to *C. jejuni* mainly occurs by the consumption of poultry meat contaminated during processing [6]. Throughout the farm-to-fork continuum of poultry products, *C. jejuni* can increase in number only in the gastrointestinal tracts of poultry due to the inability of *C. jejuni* to grow outside the host.

The pre-harvest control of *Campylobacter* critically influences poultry contamination at harvest and post-harvest levels, and consequently human exposure to *Campylobacter*. Quantitative microbial risk assessment studies evaluated that a 1–2 log reduction in the level of *Campylobacter* in poultry intestines may decrease the risk of campylobacteriosis associated with poultry consumption by 44% and 95% [7]. A 2-log reduction of *Campylobacter* counts on chicken carcasses would decrease human campylobacteriosis by 30-fold [8]. Vaccination is considered a potential intervention strategy to control *Campylobacter* at the pre-harvest level. Several vaccine types–such as killed cell lysates, subunit vaccines using recombinant proteins, and bacterial vector-based vaccines–have been tested to control *Campylobacter* in poultry. Compared to killed or recombinant vaccines, live-attenuated vaccines may offer many advantages in treating enteric pathogens, such as the presentation of various antigens and the effective stimulation of host immune systems [9]. Despite these advantages, currently, little has been done to develop live-attenuated oral vaccines to control *Campylobacter*.

*C. jejuni*, as a microaerophile, requires low concentrations of oxygen but is sensitive to oxygen in the atmosphere [10,11]. Bacterial metabolism utilizing oxygen unavoidably produces toxic reactive oxygen species (ROS) as byproducts [12]. *C. jejuni* possesses only a single copy of genes encoding ROS-detoxification enzymes, including superoxide dismutase (SodB), catalase (KatA), and alkyl hydroperoxide reductase (AhpC) [13], whereas most other bacteria carry redundant copies of these oxidative stress defense genes [12,14]. These ROS-detoxification enzymes play a critical role in the survival of *C. jejuni* under aerobic conditions [15,16] and also significantly contribute to virulence and chicken colonization [17,18,19]. Studies have shown that knockout mutants of these oxidative stress defense genes significantly compromise *C. jejuni* colonization of chicken intestines [20,21]. *ΔkatA* and *ΔsodB* mutants are defective in the colonization of chicken intestines [21], and the *ΔahpC* mutant demonstrates attenuated colonization by 50,000-fold in *C. jejuni* NCTC 11168 [21]. Based on the previous studies, we hypothesized that the pre-colonization of chicken intestines by these oxidative stress defense mutants may competitively exclude colonization by campylobacters from the environment and will be excreted from chickens due to their impaired colonization ability. In aerobic environments, the mutants cannot survive because of increased sensitivity to oxygen. In this proof-of-concept study, we evaluated the effects of the pre-colonization of chickens by oxidative stress defense mutants on the prevention of colonization by *C. jejuni* in order to identify gene targets with which to develop live-attenuated oral vaccines.

## 2. Materials and Methods

### 2.1. Bacterial Strains and Culture Conditions

*C. jejuni* NCTC 11168 and its isogenic knockout mutants of *ahpC*, *katA*, and *sodB*, which were constructed in our previous studies [22,23], were used in this study. The purpose of this study was to identify genes appropriate for developing live-attenuated *Campylobacter* vaccines. Thus, oxidative stress defense mutants constructed with an antibiotic resistance marker were used to monitor their colonization levels easily. *C. jejuni* strains will be grown at 42 °C on Mueller-Hinton (MH) media in a microaerobic condition (5% O_2_, 10% CO_2_, 85% N_2_). Kanamycin (50 μg/mL) will occasionally be added to culture media where required.

### 2.2. Evaluation of Efficacy in Preventing Campylobacter Colonization by Pre-Colonization with Oxidative Stress Defense Mutants

Chicken colonization experiments were conducted at the poultry research facility, Faculty of Veterinary Science, Chulalongkorn University, according to the animal use protocol number 1431092, which was reviewed and approved by Chulalongkorn University Animal Care and Use Committee.

A total of 99 day-of-hatch commercial broiler chicks (Cobb 500) were used in this study. In the first experiment, 55 day-of-hatch commercial broiler chicks were randomly divided into five groups including three treatment groups (*ΔahpC*, *ΔkatA*, and *ΔsodB* mutants), a positive control group, and a negative control group. The absence of *Campylobacter* in chicks was confirmed by culturing cloacal swabs on MH agar plates containing *Campylobacter*-selective supplements (SR0232E and SR0117E; Oxoid). At three days old, chicks in each treatment group were orally challenged with 0.5 mL of *C. jejuni* mutant strain grown in fresh MH broth at the concentration of ca. 1 × 10^8^ CFU/mL. These birds were later challenged with 0.5 mL of MH broth containing the wild-type *C. jejuni* strain NCTC 11168 (WT) at the concentration of ca. 1 × 10^8^ CFU/mL at 10 days of age. Cloacal swab sampling was performed weekly until the birds were 42 days old. Fecal samples were plated on MH agar plates containing *Campylobacter*-selective supplements to count the total *C. jejuni*. *C. jejuni* mutant strains were enumerated by culturing on MH agar plates supplemented with *Campylobacter*-selective supplements and kanamycin. A positive control group was challenged only with WT at 10 days of age, whereas chicks in a negative control group were treated only with PBS.

In the second experiment, two concentrations of *ΔahpC* mutant strain were further evaluated for their efficacy in preventing the colonization by WT *C. jejuni*. In the first treatment group, 11 day-of-hatch commercial broiler chicks were orally challenged with 0.5 mL of *ΔahpC* mutant at the concentration of ca. 1 × 10^9^ CFU/mL at three days of age and then challenged with 0.5 mL of WT at the concentration of ca. 1 × 10^8^ CFU/mL at 10 days of age. In the second treatment group, another 11 day-of-hatch broiler chicks were orally challenged with 0.5 mL of *ΔahpC* mutant at the concentration of ca. 1 × 10^7^ CFU/mL at three and seven days of age prior to receiving WT at 14 days of age. Positive and negative control groups (11 birds per group) were orally challenged with WT and PBS, respectively, when the birds were 14 days old. Fecal sample collection and enumeration of both total *C. jejuni* and *ΔahpC* mutant strains were performed as previously described in the first experiment.

## 3. Results

### 3.1. Prevention of C. jejuni Colonization by Pre-Colonization with Oxidative Stress Defense Mutants

**Figure 1:**
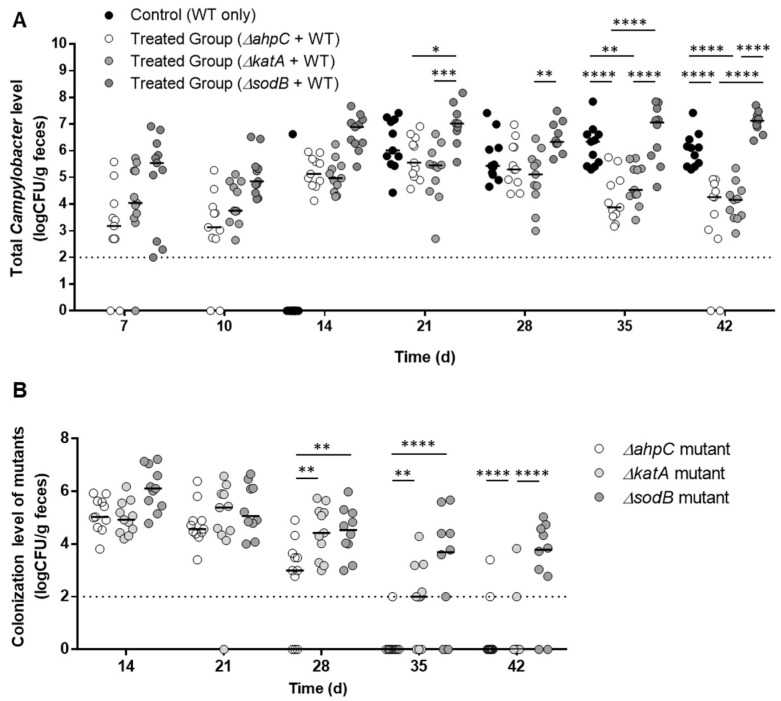
Assessment of *C. jejuni* reduction in chickens by pre-colonization with oxidative stress defense mutants, including *ΔahpC*, *ΔkatA*, and *ΔsodB***.** The figures show the colonization levels of total *C. jejuni*, including WT and the mutants (**A**), and mutants only (**B**). Day-old chicks were administered with the mutants on day three and challenged with wild type (*C. jejuni* NCTC 11168) on day 10. Median values are indicated with lines. The data were analyzed with two-way ANOVA followed by Tukey’s multiple comparison test (*: *p* < 0.05, **: *p* < 0.01, ***: *p* < 0.001, and ****: *p* < 0.0001). The dotted line is the detection limit.

The level of *C. jejuni* colonization in chickens was measured after pre-colonization with *ΔahpC*, *ΔkatA*, and *ΔsodB* mutants. Day-old chicks were orally challenged with *C. jejuni* strains. WT (i.e., *C. jejuni* NCTC 11168) colonized the gastrointestinal tract of chicks at a level of approximately 6 log_10_CFU/g feces within 21 days (Figure 1A). Until 28 days, the colonization levels were not different between the vaccinated groups and a non-vaccinated control: however, the colonization levels of total *C. jejuni*–including WT and the mutants–in the chickens pre-colonized with *ΔahpC* and *ΔkatA* were significantly reduced by 2.7 log_10_CFU/g feces and 2 log_10_CFU/g feces, respectively, after 42 days, compared to the non-vaccinated control (Figure 1A). Interestingly, the *ΔahpC* and *ΔkatA* mutants were excreted from chickens within 42 days (Figure 1B). In contrast, pre-colonization with the *ΔsodB* mutant increased the level of total *C. jejuni* after 42 days and remained in chickens at a level of 3.2 log_10_CFU/g feces after 42 days (Figure 1A). These results suggest that *ahpC* and *katA* can be potential targets for the development of live-attenuated *Campylobacter* vaccines.

### 3.2. C. jejuni Reduction by Challenge with the ΔahpC Mutant A High Dose

**Figure 2:**
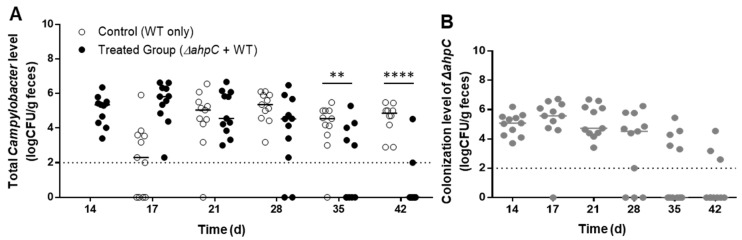
Efficacy of preventing *C. jejuni* colonization in chickens by exposure to a high dose of the *ΔahpC* mutant. (**A**) Levels of chicken colonization of the total *C. jejuni* including the wild type and the *ΔahpC* mutant. (**B**) Colonization levels of the *ΔahpC* mutant. Day-old chicks were administered with a high dose (5 × 10^8^ CFU/bird) of the *ΔahpC* mutant on day three and challenged with the wild-type *C. jejuni* on day 10. Median values are indicated with lines. Statistical significance was calculated with two-way ANOVA with Sidak’s multiple comparison test (**: *p* < 0.01, ****: *p* < 0.0001). The dotted line is the detection limit.

Previous studies conducted by our laboratory and others reported that oxidative stress defense mutants, particularly the *ΔahpC* mutant, are significantly defective in survival under aerobic conditions [15,16]. Based on the compromised aerotolerance of the *ΔahpC* mutant, the significant effect on *C. jejuni* colonization (Figure 1A), and the rapid clearance from chickens (Figure 1B), the *ΔahpC* mutant was selected for further experiments to characterize the effect of dosage on *Campylobacter* levels. Chicks were orally treated with a higher dose (ca. 5 × 10^8^ CFU/bird) than what was used in the first experiment. When a high dose was used, vaccine efficacy was significantly increased, reducing the levels of total *C. jejuni* by 2.2 and 3.9 log_10_CFU/g on 35 and 42 days, respectively (Figure 2A). When the dose was increased, the *ΔahpC* mutant colonized the chicken intestines at around 5 log_10_CFU/g for three weeks and was substantially removed from chickens after 42 days (Figure 2B).

### 3.3. C. jejuni Reduction by Challenge with the ΔahpC Mutant A Low Dose

**Figure 3:**
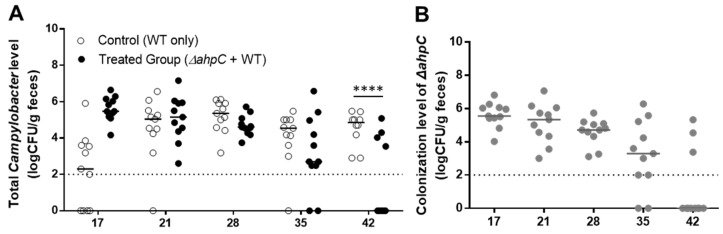
Efficacy of preventing *C. jejuni* colonization in chickens by two exposures to a low dose of the *ΔahpC* mutant. (**A**) Levels of chicken colonization of the total *C. jejuni* including the wild type and the *ΔahpC* mutant. (**B**) Colonization levels of the *ΔahpC* mutant. Day-old chicks were administered with a low dose (5 × 10^6^ CFU/bird) of the *ΔahpC* mutant on days three and seven and challenged with the wild-type *C. jejuni* on day 10. Median values are indicated with lines. Statistical significance was calculated with two-way ANOVA with Sidak’s multiple comparison test (****: *p* < 0.0001). The dotted line is the detection limit.

After examining the efficacy in *C. jejuni* reduction by the *ΔahpC* mutant at a high dose, chicks were treated with a lower dose (ca. 5 × 10^6^ CFU/bird) of the *ΔahpC* mutant twice on days three and seven. Oral challenge with the *ΔahpC* mutant at a low dosage reduced the levels of *C. jejuni* colonization by 3 log_10_CFU/g after 42 days compared to the levels in the untreated control (Figure 3A). The levels of colonization by the *C. jejuni ΔahpC* mutant increased until 17 days and started decreasing after 21 days (Figure 3B). In contrast, when chickens were challenged with a low dose only once, there was no effect on *C. jejuni* colonization (data not shown), suggesting when a low dose is used, chickens should be exposed to the *ΔahpC* mutant at least twice to achieve significant *C. jejuni* reduction.

### 3.4. Increase in Chicken Body Weight after Pre-Colonization with the Δahpc Mutant

Compared to the control group that was treated with only WT, the pre-colonization with the *ΔahpC* mutant increased the average body weight of chickens on day 42. When chickens were challenged with a dose of 5 × 10^8^ CFU/bird, the average weight of chickens in the treated group increased by 8.8% on day 42 compared to the control (Table 1). In another experiment, the average body weight of chickens on day 42 was increased by 3.2% with a low dose (ca., 5 × 10^6^ CFU/bird) challenge twice and 4.4% after treatment with a high dose (ca., 5 × 10^8^ CFU/bird) and (Table 1). However, the differences were not statistically significant. Nevertheless, these results suggest that the pre-colonization of chickens with the *ΔahpC* mutant may increase the overall growth of chickens.

## 4. Discussion

When compared to the common types of vaccines, such as killed or subunit vaccines, live-attenuated oral vaccines have multiple advantages in treating enteric pathogens. For instance, live-attenuated oral vaccines are resistant to enzymatic degradation in the host intestines, whereas recombinant subunit vaccines should confront proteolytic degradation in the gastrointestinal tracts [24]. Killed vaccines are relatively less effective in stimulating an immune response and require an adjuvant for vaccination. For instance, subcutaneous vaccination with formalin-killed *C. jejuni* using oil and aluminum hydroxide gel as adjuvants did not reduce the level of *C. jejuni* in chickens despite significant induction of anti-*Campylobacter* antibodies [25]. Oral vaccination with formalin-inactivated *C. jejuni* using *Escherichia coli* heat-labile toxin as an adjuvant increased the level of anti-*C. jejuni* secretory IgA but resulted in only a limited reduction in the *C. jejuni* level by 1.4 log CFU/g feces [26].

Live-attenuated vaccines can present various antigens and boost host immune systems more effectively than killed vaccines [9]. Whereas recombinant and killed vaccines require purification and inactivation processes, the production of live-attenuated bacterial vaccines is easy and less expensive. Regardless of the vaccine type and efficacy, production costs should be affordable for the poultry industry. Furthermore, live-attenuated oral vaccines can be used by adding them to drinking water, which makes vaccination convenient. Studies thus far to develop live-attenuated oral *Campylobacter* vaccines adopted delivery systems using other bacteria to express *Campylobacter* antigens [27,28], because *Salmonella*- and *E. coli*-based delivery systems have been technically well established. Oral vaccination with live-attenuated *Salmonella* vaccines expressing the *C. jejuni* amino acid binding protein CjaA induced CjaA-specific serum IgY and biliary IgA, and reduced *C. jejuni* colonization by about 1.4 log CFU/g in cecal contents of vaccinated chickens [29]. However, such an approach has some limitations. First, *C. jejuni* glycosylates proteins [30]; since protein glycosylation promotes an immunomodulatory function in *C. jejuni* [31], live-attenuated *Campylobacter* vaccines using other bacteria cannot express antigenic glycosylated proteins without specific genetic engineering to introduce the genes for protein glycosylation into the bacteria [32]. If live-attenuated vaccines can be developed using *Campylobacter*, glycosylated antigenic proteins can increase vaccine efficacy.

Ideally, live-attenuated oral *Campylobacter* vaccines should be able to colonize the gastrointestinal tract of a bird within a couple of weeks of age because chicks are naturally colonized by *Campylobacter* within 2–3 weeks after hatching through horizontal transmission from environmental sources [33]. When we first planned this project, maternal antibodies were a concern. Due to the common prevalence of *Campylobacter* in chickens, *Campylobacter*-specific maternal antibodies can prevent *Campylobacter* colonization in young (<two weeks) chicks [34], although *Campylobacter* infection significantly increases at three to four weeks of age when maternal antibodies disappear [35]. However, our results showed that oxidative stress defense mutants could colonize chicks successfully (Figure 1).

**Figure 4:**
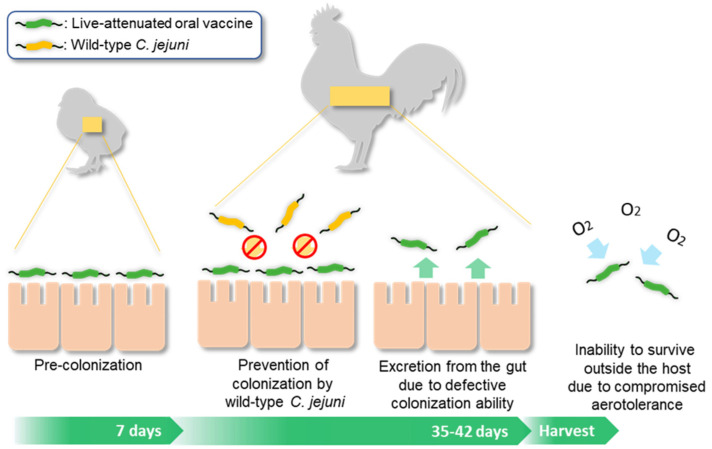
Rationale for how the *ΔahpC* mutant works as a live-attenuated oral vaccine.

The results of this study demonstrated that pre-colonization by the *ΔahpC* mutant is effective at excluding *Campylobacter* from poultry, which is the most important function to be played by a live-attenuated oral vaccine. Notably, *C. jejuni* reduction was significant when chickens were orally challenged with a high dose (ca., 5 × 10^8^ CFU/bird) of the *ΔahpC* mutant or a low dose (ca., 5 × 10^6^ CFU/bird) twice (Figure 2 and Figure 3). Since it is practically impossible to vaccinate each bird using a syringe on farms, we expect that birds should be treated with live-attenuated oral vaccines in drinking water. In this case, the dosage cannot be precisely controlled for each bird. For this, our results suggest that the *ΔahpC* mutant can exclude *C. jejuni* whether chickens are exposed to either a high dose only once or a low dose at least twice within seven days before exposure to campylobacters from the environment. After competitively preventing colonization by *Campylobacter* from the environment, a live-attenuated oral vaccine should be excreted from a bird and should not survive outside the host. The oxidative stress defense mutants can meet this requirement. Particularly, the *ΔahpC* mutant shows compromised aerotolerance and easily loose viability in aerobic environments [15,16] (Figure 4).

In this proof-of-concept study, we aimed to validate the effects of pre-colonization by oxidative stress defense mutants on preventing colonization by WT *C. jejuni* and to identify gene targets for the development of live-attenuated *C. jejuni* vaccines. Our data based on *C. jejuni* colonization suggest that the *ΔahpC* mutant has a great potential for on-farm application to control *C. jejuni* at the pre-harvest level. Possibly, pre-colonized *ΔahpC* mutant can competitively exclude the colonization by WT as they may compete for colonization sites and nutrients. Although we did not measure immune response in chickens after oral challenge with the *ΔahpC* mutant in this study, we can speculate that similar or the same immune responses will be induced in chickens as induced by WT *C. jejuni* because the mutant is a live *C. jejuni* strain defective with only a single gene, which can colonize the gastrointestinal tract of chickens (Figure 1, Figure 2 and Figure 3). In chickens, *C. jejuni* colonization results in a significant increase in anti-*Campylobacter* serum IgY and bile IgA [36], and induces pro-inflammatory responses [37,38]. One of the challenges in *Campylobacter* control using vaccination is the short life span of commercial broilers, which is usually about several weeks depending on the body weight. Whereas the avian innate immune system matures rapidly in response to challenges with enteric bacteria, the adaptive immune response in the gut in chickens begins to mature at six weeks of age [39]. This means that chickens can limitedly develop immune responses even to vaccination in the course of broiler production. Although our present study does not provide data about the immune responses after pre-colonization by the *ΔahpC* mutant, the significant reduction in the level of *C. jejuni* colonization indicates the approach of this study is feasible and has potential for practical application.

In addition to immune response induction, the pre-colonization of the *ΔahpC* mutant may also affect the gut microbiota. If chickens are colonized by *C. jejuni* at an early age, such as 6 days of age, the microbiota change is persistent, and even changes in the cecal microbiota made by late colonization still endure in chickens ready for market, indicating that *C. jejuni* colonization makes a substantial effect on the cecal microbiota [38]. Based on these previous studies, the early colonization of chickens by the *ΔahpC* mutant may affect both immune responses and gut microbiota in chickens.

The live-attenuated oral vaccine developed in this study has additional features suitable for application to poultry production. In addition to a notable reduction of *C. jejuni* levels within the time for broiler production (Figure 1A, Figure 2 and Figure 3), the pre-colonization by the *ΔahpC* mutant increased the average body weight of chickens on day 42 compared to the control group treated with WT (Table 1). Although the differences in body weight were not statistically significant, the average increase in the body weight of chickens may increase productivity. At this point, we cannot explain the mechanism behind the body weight increase by oral administration with the *ΔahpC* mutant. It has been suggested that the induction of pro-inflammatory responses by *C. jejuni* colonization may reduce the body weight of chickens by disrupting nutrient absorption [36,37,38]. We observed that the body weight of chickens treated with PBS was always higher than that of those challenged with only WT (Table 1), suggesting *C. jejuni* colonization can affect the body weight of chickens. In our study, each experimental group was separated, and chickens treated with only PBS did not have the chance of environmental exposure to *C. jejujni* and remained negative for *C. jejuni*, which is different from real farm conditions. Since broilers can carry high levels of *Campylobacter* (ca., 10^6^~10^9^ CFU/g feces) [40], chickens in the group treated with WT can be similar to broilers on farms based on the level of *Campylobacter*. Presumably, the reduction of *C. jejuni* by pre-colonization with the *ΔahpC* mutant may result in body weight increase by alleviating pro-inflammatory immune responses because the increase was seen in after day 21 (i.e., day 42 in Table 1) when the level of *C. jejuni* colonization was reduced (Figure 1A, Figure 2 and Figure 3). In addition, alterations in gut microbiomes by pre-colonization with the *ΔahpC* mutant can also affect body weight. However, the hypothesis awaits future validation.

Live-attenuated *Campylobacter* vaccines reported to date, including those presented in this study, are not suitable for obtaining government approval because they were constructed using genetic modifications with antibiotic resistance markers. To construct a live-attenuated oral vaccine applicable for poultry farms, we developed a new in-frame deletion metagenesis method that does not leave any antibiotic resistance markers in a vaccine construct, which is different from the previously reported in-frame deletion method that uses streptomycin-resistant *C. jejuni* [41]. Using the new method, we constructed an in-frame deletion mutant of *ahpC* that does not have an antibiotic resistance marker and discovered that this *ΔahpC* mutant made similar effects on preventing *Campylobacter* colonization in chickens (data not shown). Our data based on the level of *C. jejuni* colonization suggest that the *ahpC* mutant has a great potential for on-farm application to control *C. jejuni* at the pre-harvest level, which is a novel one health-based approach to reduce human exposure to *Campylobacter*. Future studies will examine how the pre-colonization of the *ΔahpC* mutant affects immune responses and gut microbiome to elucidate mechanisms for the reduction in *C. jejuni* colonization.

## 5. Conclusions

In this proof-of-concept study to develop live-attenuated *C. jejuni* vaccines using oxidative stress defense mutants, we discovered that pre-colonization of chickens with a mutant defective in *ahpC* significantly reduced the level of *C. jejuni* and increased body weights in chickens. The *ahpC* gene is a potential target for the construction of live-attenuated *C. jejuni* vaccines for chickens.

## 6. Patents

The findings of this study have been filed for a United States provisional patent.

## Figures and Tables

**Table 1:** Effects of pre-colonization with the *ΔahpC* mutant on body weight increase in chickens.

Day		0 d	21 d	42 d
**1st Experiment**	Control (PBS; *n* = 11)	47.06 ± 2.92 g	890.36 ± 70.29 g	2114.55 ± 204.76 g
	Control (WT; *n* = 11)	44.09 ± 3.40 g	981.00 ± 104.05 g	2101.50 ± 363.49 g
	Treated group (*ΔahpC* + WT; *n* = 11) ^1^	45.91 ± 3.02 g	993.18 ± 91.17 g	2289.55 ± 246.95 g
**2nd Experiment**	Control (PBS; *n* = 11)	26.27 ± 4.45 g	980.00 ± 27.93 g	2400.00 ± 163.30 g
	Control (WT; *n* = 11)	38.64 ± 6.36 g	940.00 ± 84.38 g	2150.00 ± 190.03 g
	Treated group (*ΔahpC* + WT; *n* = 11) ^2^	42.91 ± 6.01 g	910.91 ± 126.29 g	2218.18 ± 348.76 g
	Treated group (*ΔahpC* + WT; *n* = 11) ^3^	39.55 ± 4.72 g	906.36 ± 60.71 g	2245.45 ± 136.85 g

The dosages of the *ΔahpC* mutant were ca. 5 × 10^7^ CFU/bird ^1^, 5 × 10^6^ CFU/bird (two challenges days 3 and 7) ^2^, and 5 × 10^8^ CFU/bird ^3^.

## Data Availability

Not applicable.
